# Time Hierarchies and Model Reduction in Canonical Non-linear Models

**DOI:** 10.3389/fgene.2016.00166

**Published:** 2016-09-21

**Authors:** Hannes Löwe, Andreas Kremling, Alberto Marin-Sanguino

**Affiliations:** Specialty Division for Systems Biotechnology, Technische Universität MünchenGarching, Germany

**Keywords:** mathematical modeling, biochemical systems theory, quasi-polynomial systems, time-scales, model reduction, systems biology

## Abstract

The time-scale hierarchies of a very general class of models in differential equations is analyzed. Classical methods for model reduction and time-scale analysis have been adapted to this formalism and a complementary method is proposed. A unified theoretical treatment shows how the structure of the system can be much better understood by inspection of two sets of singular values: one related to the stoichiometric structure of the system and another to its kinetics. The methods are exemplified first through a toy model, then a large synthetic network and finally with numeric simulations of three classical benchmark models of real biological systems.

## 1. Introduction

Biochemical systems are amenable to be modeled using differential equations but, due to the great diversity of mechanisms involved, the resulting models lack a defined structure. There is seldom a common set of properties that might simplify their analysis or enable the development of general tools. Models with a well defined structure enable a great level of abstraction and generality. Control engineering using linear systems is a case in point, where chemical plants, steam engines, and electric systems can all be treated within the same framework. In opposition to that, the analysis of *ad-hoc* biological models is often restricted to the numerical integration of a few scenarios.

The difficulties to analyze biological systems start with identification of which components to include in—or exclude from—the model, since the cellular milieu contains many, highly interconnected components. In addition to that, the intervening processes often progress at different time-scales, the resulting models tend to be stiff and difficult to analyze. But multiple time-scales also offers an opportunity for a deeper analysis (Hek, 2010). Many properties of biochemical systems are tied to the time hierarchy of the system. For instance, a regulatory mechanism must be as fast or faster that the process it is supposed to stabilize while some types of oscillations come from the interaction between a fast and a slow subsystem. An analysis of the first case need only take into account the subsystem that corresponds to the right time-scale, while the second case would better be analyzed by focusing on interactions between a fast and a slow subsystem. Furthermore, separating time scales reduces the stiffness of the system, and the computing power needed for numerical integration of the models.

A wide variety of time scale separation methods is available (Gerdtzen et al., 2004; Jamshidi and Palsson, 2008) but no single all-round solution has been found due to the difficulties associated to the diversity of biological models and their non-linearity. Many model reduction methods are based in rewriting the model—e.g., through non-dimensionalization—in a form in which the different time-scales are shown explicitly

(1) $$\begin{array}{lll} ẋ=\;\varepsilon f\left(x,y,t\right),\; & x\left(0\right)=x_{0}\;\\ ẏ=g\left(x,y,t\right),\; & y\left(0\right)=y_{0}\; & \; \end{array}$$

since the derivative of *x* is multiplied by the small perturbation parameter ϵ, it will have slower dynamics than *y*. This is a regular perturbation problem for which an approximate solution can be obtained by writing the equations when ϵ → 0. The solution of:

(2) $$\begin{array}{lll} ẋ=\;0,\; & x\left(0\right)=x_{0}\;\\ ẏ=g\left(x,y,t\right),\; & y\left(0\right)=y_{0}\; & \; \end{array}$$

describes the fast dynamics of the system for intervals of time small enough that the change in *x* is negligible. The solution to this simplified problem is called inner solution and it is generally valid for the thin time slice *t* = O(ε). In order to obtain the slow dynamics, a reparametrization τ = ε*t* can be performed to obtain:

(3) $$\begin{array}{l} \;\frac{dx}{d\tau }=\;f(x,y,\tau ),\;x(0)=x_{0}\\ \varepsilon \frac{dy}{d\tau }=\;g(x,y,\tau ),\;y(0)=y_{0} \end{array}$$

Which is a singular perturbation problem, since the order of the equations changes when ε → 0, yielding.

(4) $$\begin{array}{l} \frac{dx}{d\tau }=\;f(x,y,\tau ),\;x(0)=x_{0}\\ \;0=\;g(x,y,\tau ) \end{array}$$

Since the original differential equation for *y* becomes an algebraic equation, its initial condition *y*(0) = *y*_0_ can no longer be satisfied. However, provided that the eliminated equation for $\dot{y}$ had a hyperbolic solution (one lacking a central manifold), this approximation will be valid for *t* ≫ ε and it is also known as the outer solution.

These kinds of problem fall within the category known as boundary layer problems, alluding to the transition between the inner and the outer solution. Obtaining a uniformly valid solution for all times, requires the matching of the inner and outer solution, however, when one is interested in the behavior of the system well within the area of validity of each solution, as is the case in biology, the inner and outer solutions are informative enough.

The appearance of algebraic equations introduces difficulties of its own. When the solution of Equation (2) can be written explicitly, *y* = ϕ(*x, t*), then algebraic constants can be eliminated by back-substitution in the o.d.e.s:

(5) $$\begin{array}{ll} ẋ=\;f\left(x,\phi \left(x,t\right),t\right),\;x\left(0\right)=x_{0}\; & \; \end{array}$$

Finding this solution and substituting it as well as the non-dimensionalization step itself are no easily accomplished for big non-linear systems. The wide variety of possible structures for the equations is a challenge for any attempt to do this systematically.

## 2. Materials and methods

### 2.1. Modal analysis

The advantage of dealing with a system that has a regular, convenient structure is made evident by analyzing time scales in the linear case. It has been shown (Palsson et al., 1987; Jamshidi and Palsson, 2008) that linearizing around a certain steady state and decomposing the Jacobian matrix of the system, allows to define aggregate variables or modes:

(6) $$J=M^{-1}\Lambda M$$

where **Λ** is a diagonal matrix with the eigenvalues of **J**. Some eigenvalues/eigenvectors may be imaginary conjugates, in that case, a similar decomposition may be used where **Λ** will be a Jordan canonical form. In any case, new variables can be defined:

(7) m=Mx

and the linearized differential equation would be:

(8) $$\dot{m}=\Lambda m$$

Since **Λ** is diagonal, each mode *m*_*i*_ will vary independently of the rest in the linearized system, except for modes corresponding to conjugate pairs of eigenvalues, which will remain bound together. Modes enable to find combinations of variables with different timescales even for cases when the time scales of all the variables are similar. Modes work ideally with linear systems since the modes themselves are linear combinations of the variables. Back-substituting linear expressions in a linear system does not alter its structure, because of the telescopic property. For a non-linear system, however, the Jacobian matrix changes in every point so the modes will only be uncoupled at the point where the system is linearized. furthermore, non-linear systems do not normally comply with the above mentioned telescopic property, which results in differential-algebraic systems.

In subsequent sections we will apply these and similar concepts to a very general class systems that, in spite of being non-linear, have a regular structure and some extremely useful properties.

### 2.2. Canonical non-linear forms

The theoretical results of this work arise from the properties of the power-law and quasi-polynomial formalisms. These two formalisms have been shown to be mathematically equivalent. Whether a model is simpler in one mechanism or another depends on the particular processes involved. In general, power-law models are preferred to describe processes that depend on absolute fluxes (e.g., chemical networks) and quasi-polynomial models are used for modeling processes based on *per capita* rates, like logistic equations or classical predator-prey models. In any case, any system of differential equations that fulfills one of the formalisms can be rewritten to the other without any loss of information. Furthermore, virtually any non-linear system of differential equations can be rewritten as one of the above mentioned systems through approximation (Savageau, 1969a), detailed mechanistic representation (Savageau, 1998), exact recasting (Savageau and Voit, 1987), or partitioning of its parameter space (Savageau et al., 2009; Lomnitz and Savageau, 2015).

#### 2.2.1. Power-law

The most general expression for these models is as linear combinations of rates or fluxes:

(9) $$\dot{x}=Nv(x)$$

where each term $v_{i}=\gamma_{i}\prod_{j}x_{j}^{f_{i,j}}$ are the power-laws the formalism takes the name from. The rate constants, γ_*i*_ are positive real numbers and the kinetic orders, *f*_*i, j*_, are real numbers normally between −2 and 2. It is also common to include “inputs” to the system as the so called independent variables, that reflect the environment in which the system operates and remain constant during a simulation or experiment. These variables can be included as part of γ without loss of generality. This kind of model is called Generalized Mass Action (GMA). All the formalisms discussed here can be expressed in a very convenient form adopting a direct notation proposed by Lewis Voit (1991) and that is slowly being adopted for theoretical analyses involving power-laws (Marin-Sanguino et al., 2010; Müller et al., 2016). So GMA equations becomes:

(10) $$\dot{x}=N\text{ diag}(\gamma )x^{F}$$

where the notation diag (·) will be used to represent a diagonal matrix containing vector (·) as its main diagonal. All the information on the system is summarized in two matrices and a vector: **N** of size *n* × *m* reflects the stoichiometry of the system—mass conversion/conservation—**F** has size *m* × *n* and contains the kinetic information. The *m* × 1 sized vector, γ, serves as a reference connecting rates and metabolites—e.g., when the system variables are normalized by their value at a certain equilibrium point, *z*_*i*_ = *x*_*i*_/|*x*|_0_, then the vector of rate constants becomes the vector of steady state fluxes of the system. Under such conditions, the partition of information becomes clear between a stoichiometric/static-flux information **N** diag(γ) and kinetics **F** a particular type of gma models, the s-systems, have received an exceptional deal of attention due to their remarkable properties. An s-system has a single positive and a single negative term:

(11) $$\dot{x}=\text{diag}(\alpha )x^{G}-\text{diag}(\beta )x^{H}$$

where **α** and **β** are rate constants and **G** and **H** are kinetic order matrices. these systems have analytic solutions for their steady states (Savageau, 1969b).

The variables in a s-system can be normalized using their steady state values (Savageau, 1974). Defining new variables $z_{i}=\frac{x_{i}}{|x_{i}{|}_{0}}$ where the zero subindex indicates the numerical value of the variable in the steady state, and rearranging terms, results in the system:

(12) $$\dot{z}=\text{diag}(f)\left(z^{G}-z^{H}\right)$$

Due to this normalization, the new variables will reach the steady state at *z*_*i*_ = 1∀*i*. The factors *f*_*i*_, are the turnovers of their respective variables at the steady-state (Savageau, 1974),

(13) $$f_{i}={\left|\frac{V_{i}^{+}}{x_{i}}\right|}_{0}={\left|\frac{V_{i}^{-}}{x_{i}}\right|}_{0}$$

and considered to contain information relative to the time scale of the corresponding variable. Actually *F*-values are the reciprocals of transition times as defined by Easterby (1981).

#### 2.2.2. Quasi-polynomial

In their more general form, quasi-polynomial systems can be written as Generalized Lotka–Volterra (GLV)

(14) $$\dot{x}=\text{diag}(x)\left(\lambda +Ax^{B}\right)$$

with **A**, **B**, and **λ** of size *n* × *m*, *m* × *n*, and *n* × 1. Just like before, the stoichiometric information is contained in one matrix and the kinetics in another.There is also a famous particular case of this kind of system, for **B** = **I**, Equation (14) becomes the Lotka–Volterra model for *n* species.

An important property of GLV systems is their invariance when subject to quasimonomial transformations **x** = **y**^**C**^, where **C** is a square non-singular matrix. The result of this transformation is a GLV system itself:

(15) $$\dot{y}=\text{diag}(y)\left(\hat{\lambda }+\hat{A}y^{\hat{B}}\right)$$

where

(16) $$\begin{array}{l} \hat{A}=C^{-1}A\\ \hat{\lambda }=C^{-1}\hat{\lambda }\\ \hat{B}=\;BC \end{array}$$

All the systems that can be converted into one another through a quasimonomial transformation form a class of equivalence, sharing a great deal of important properties such as number of steady states and their stability (Hernández-Bermejo and Fairén, 1995).

A very complete account of the properties of this formalism can be found in Hernández-Bermejo et al. (1998), but we will describe two applications of the quasimonomial transformation, that are specially relevant in this context.

When matrix **B** does not have full rank *r* < *n*, a transformation matrix can be chosen

(17) $$C=\left(\begin{array}{c} I_{r\times r}\\ 0_{n-r\times r} \end{array}\begin{array}{cc} \phi_{1}\ldots & \phi_{k} \end{array}\right)$$

where **ϕ_*i*_** *i* = 1…*k* are basis vectors for the kernel of **B**. The transformed exponents will in this case be $\hat{\text{B}}=[B_{m\times r}|0_{m\times k}]$. From the structure of Equation (14), it follows that a number of variables in the transformed system equal to the dimension of ker(**B**) have no influence on any equation other than their own. These variables result in quadratures and can therefore be taken out of the system.

Any GLV can be converted to a Lotka–Volterra as a special case of quasi-monomial transformation **q** = **x**^**B**^, that results in the Lotka–Volterra model where the variables are replaced by the quasi-monomial terms:

(18) $$\dot{q}=\text{diag}(q)\left(B\lambda +BAq\right)$$

Since the number of quasi-monomial is often greater than that of variables, matrix **B** is seldom square and **BA** will often be singular. From Equation (16) follows that any two systems from a class of equivalence will result in the same Lotka–Volterra representation, which can be taken to be a canonical form for the whole class. In the Lotka–Volterra systems, all non-linearities of the system are reduced to quadratic terms and any interaction term between two variables has the form *cq*_*i*_*q*_*j*_ where the constant *c* is the (i,j)-th entry of **BA**.

#### 2.2.3. Relation between the formalisms

Any system written as a power-law can be translated to a quasi-polynomial system and vice versa. This is trivial for small systems and can be done applying a formula to the matrices of arbitrarily large and complex systems (Marin-Sanguino et al., 2010). This similarity leads to many common properties that have been found using completely different methods in both formalisms. For instance, the symmetry matrix of an autonomous GMA or s-system (Voit, 1992) is the **B** matrix of the corresponding GLV. The rank deficiency in such matrix, implies existence of parameter transformation groups that can decouple a power-law system the same way transformation (Equation 17) does with a GLV. From now on, we will consider both formalisms to be equivalent (Voit and Savageau, 1986), so we can talk, for instance, about the **B** matrix of a GMA or the class of equivalence to which an s-system belongs.

### 2.3. Numerical simulations

To verify the theoretical considerations, we simulated different non-linear models in s-system representation that were taken from the literature (Voit, 2000).

#### 2.3.1. Integration of the differential equations with perturbed initial values

Differential equations were numerically integrated with Matlab's ode15s solver. Integration time was estimated from the the biggest real eigenvalue of the Jacobian of the linearized, full system at its steady state:

$$t_{end}=-{\left(\max\left(Re(\underline{\lambda })\right)\right)}^{-1}\cdot 5$$

The resulting trajectories of the slow variables—those not in quasi-steady-state (qss)—of the original system were compared to the trajectories of the reduced system, in which the fast variables are assumed to be in qss. The robustness of the approximation was tested by performing simulations of the full system in which the qss variables had random initial values distant to qss by a factor of 10.

The value for the absolute perturbation of the fast variables is defined as the Euclidean norm of the natural logarithm of the quotients of the initial values of the fast variables *x*_*f*, 0_ and the corresponding quasi-steady state values at time 0, *x*_*f, qss*_:

$$\delta y=\left\|\left(\begin{array}{c} ln\left(\frac{x_{f_{1},0}}{x_{f_{1},qss}}\right)\;ln\left(\frac{x_{f_{2},0}}{x_{f_{2},qss}}\right)\;\cdots \;ln\left(\frac{x_{f_{n},0}}{x_{f_{n},qss}}\right) \end{array}\right)\right\|$$

with n fast variables *x*_*f*_*i*__.

#### 2.3.2. Effect of the perturbation of fast variables on the slow trajectories

In a next step, the effect of this perturbation was tested. The data of the slow trajectories was sampled at defined times for the full and the reduced system and the mean and standard deviation at these points in time were calculated for 1000 simulations with randomized initial values.

Additionally the trajectory of the slow variables in the original, full system was also interpolated and sampled at the same points in time. To get an objective measure of the relative error between reduced and full system, first the relative error of the trajectories in the reduced system *x*(*t*)_*s*_*i*_, *qssa*_ compared to the full system *x*(*t*)_*s*_*i*__ was calculated for each of the slow variables by:

$$E_{i}=\frac{\int_{0}^{t_{end}}|x{\left(t\right)}_{s_{i},qssa}-x{\left(t\right)}_{s_{i}}|dt}{\int_{0}^{t_{end}}|x{\left(t\right)}_{s_{i}}|dt}$$

with *x*_*s*_*i*_, *ss*_ being the steady-state-values that serve as a baseline for the comparison. The integral was numerically computed with the trapezoidal method, given the data from the trajectories. To get a number for the system considering all variables, the Euclidean norm of all these errors |**E**| was plotted against δ*y*. Each of the points represents one of the 1000 simulations.

### 2.4. Random network generation

In order to benchmark the methods for large systems, synthetic genetic networks were generated. When modeled as s-systems, this networks consists of a matrix of kinetic orders and a vector of turnover numbers as indicated below in the results section. The models were generated in python using the standard libraries scipy and numpy. The turnover numbers were generated at random in three groups to ensure the existence of three different time-scales. Each group was generated following a normal distribution with different means and standard deviations calculated from the distances between the means to guarantee the existence of three distinct. The number of variables in each group (time-scale) was also predetermined. The kinetic order matrices were generated as sparse matrices of density 0.05. Each network was tested to ensure stability and that all the components were connected (using the library Network X).

## 3. Results

### 3.1. Log-modes

The Jacobian of a system under a a particular set of transformations (like the quasimonomial transformation) will always have the same eigenvalues as those of the original system. In the case of s-systems under the logarithmic transformation, the Jacobians are identical. S-systems can be explicitly rewritten after undergoing the transformation **y** = log(**x**), the transformed equations take the form (Savageau, 1976),

$$\dot{y}=\text{diag}\left(\alpha \right)\;\exp\left((G-I)y\right)-\text{diag}\left(\beta \right)\;\exp\left((H-I)y\right)$$

The Jacobian matrix of the system defined in terms of **y** at a steady state is identical to the Jacobian of the original system, so the coefficients defined in Equation (6) can be used to define a new set of modes that we will call log-modes (**ℓ**).

log(ℓ)=My

which will actually be a monomial transformation:

$$\ell =x^{M}$$

So any fast or slow manifolds identified from the log modes will take the form of a power-law and can be back-substituted into any of the formalisms here discussed without generating algebraic constraints.

### 3.2. Identifying time-scales for the variables through the s-system representation

The existence of analytic steady-state solutions in s-systems makes it possible to apply the quasi-steady-state hypothesis to obtain the behavior of the slow part of multi-level systems (Savageau, 1976; Savageau and Sorribas, 1989), a very similar procedure has been used in the context of sensitivity analysis (Delgado and Liao, 1995). In this section we will generalize the procedure to split a dynamic system into its time scales, obtaining equations for the all of them without generating algebraic constraints. We will start using the s-system representation and will then move on to more general considerations.

Without loss of generality, the variables in Equation (12) can be arranged according to their *f*-factor in decreasing order, the variables can be classified as slow or fast by finding a variable *x*_*k*_ such that ||*f*_*k*+1_ − *f*_*k*_|| is maximal. Now a non-dimensionalization for time can be applied τ = *f*_*k*_*t*

$$\frac{dz_{i}}{d\tau }=\frac{f_{i}}{f_{k}}\left(\underset{j}{\prod }z_{j}^{g_{ij}}-\underset{j}{\prod }z_{j}^{h_{ij}}\right)$$

defining $\varepsilon =\frac{f_{k+1}}{f_{k}}$, the multiplier for the first *k* equations becomes: $\frac{f_{1}}{f_{k}}>\frac{f_{2}}{f_{k}}>\cdots >1$ and the rest $\varepsilon >\varepsilon \frac{f_{k+2}}{f_{k+1}}>\cdots >\varepsilon \frac{f_{n}}{f_{k+1}}$.

(19) $$\begin{array}{l} \frac{dz_{i}}{d\tau }=\hat{f_{i}}\left(\prod_{j}z_{j}^{g_{ij}}-\prod_{j}z_{j}^{h_{ij}}\right)\;i=1,\ldots ,k\\ \frac{dz_{i}}{d\tau }=\varepsilon \hat{f_{i}}\left(\prod_{j}z_{j}^{g_{ij}}-\prod_{j}z_{j}^{h_{ij}}\right)\;i=k+1,\ldots ,n \end{array}$$

if $f_{k}>>f_{i}\Rightarrow \frac{dz_{i}}{d\hat{t}}=\epsilon \;\left(z^{G}-z^{H}\right)$. which enables to get a quasi-steady-state (qss) solution for the fast variable. s-systems share the telescopic property discussed above for linear systems so the algebraic constraints generated by the qss assumption can be back-substituted in the system as shown in the Appendix (Supplementary Material). As a result, the system can be divided in two, a fast system:

(20) $$\begin{array}{ll} \frac{dz_{i}}{d\tau }=\;\hat{\alpha_{i}}\overset{k}{\underset{j=1}{\prod }}z_{j}^{g_{ij}}-\hat{\beta_{i}}\overset{k}{\underset{j=1}{\prod }}z_{j}^{h_{ij}}\;i=1,\ldots ,k\; & \; \end{array}$$

where the slow variables are taken as constants and grouped into $\hat{\alpha_{i}}$ and $\hat{\beta_{i}}$. The normalized steady state is no longer at one, since it depends on the values assigned to the slow variables.

A time rescaling *T* = ετ provides the complementary time scale. The slow system that depends exclusively on the slow variables:

(21) $$\begin{array}{ll} \frac{dz_{i}}{dT}=\;\hat{f_{i}}\left(\overset{n}{\underset{j=k}{\prod }}z_{j}^{{ĝ}_{ij}}-\overset{n}{\underset{j=k}{\prod }}z_{j}^{{ĥ}_{ij}}\right)\;i=k+1,\ldots ,n\; & \; \end{array}$$

See Supplementary Information for a detailed calculation.

This procedure can only be applied to an s-system but it provides information of use for the more general cases. GLV systems with a single equilibrium point can be exactly rewritten as s-systems (Hernández-Bermejo and Fairén, 1995), s-systems can also dominate the dynamics of arbitrary non-linear systems in a well defined region of their parameter space (Savageau et al., 2009) or arise as good approximations through a Taylor series (Savageau, 1969a). The validity of the turnover numbers as indicators for timescales is in fact so robust, that the inverse of the turnover, the transition time, was defined as a reference in the model free setting of biochemical enzyme assays (Easterby, 1981). Turnover numbers are only a valid approach for well behaved systems in which they dominate over the rest of the equations, the next section will deal with not so well behaved systems.

### 3.3. Collinearity among the quasinomials

In order to assess whether a system is “well behaved” in the sense mentioned above, a closer examination of **B** is in order. Sensitivity to parameter combinations can be assessed through the spectrum of the corresponding matrix (Hearne, 1985). Since the matrices involved are not usually square, the SVD decomposition of the matrix, $B=U_{B}\Sigma_{B}V_{B}^{T}$, will be of great use.

It has been seen that a rank deficiency in **B** allows to decouple some of the variables of the system. This results in an invariant manifold spanned by the corresponding vectors of **V_B_** which can consist of infinite equilibrium points or preclude any sort of equilibrium (Voit, 1991). When the matrix is not singular but it is ill conditioned, a similar phenomenon happens. This can be seen by applying a quasimonomial transformation:

(22) $$x=y^{V_{B}}$$

where each of the new variables *y*_*i*_ is associated to a singular vector. The exact dynamics of all these new variables will have GLV form as shown in Equation (16). From inspection of the resulting system

(23) $$\dot{y}=\text{diag}(y)\left(\hat{\lambda }+\hat{A}y^{U_{B}\Sigma_{B}}\right)$$

it is straightforward to see that the exponents of *y*_*i*_ in all monomial terms are multiplied by σ_*i*_, as the later tends to zero, the variable will lose influence on the all the other variables, reducing the real dimension of the system.

### 3.4. The stoichiometric and kinetic components of the invariant matrix

Analyzing the log-modes of a non-linear system at a certain equilibrium point has the risk of not being representative, since its Jacobian may change dramatically when it moves away from the linear region. As has been seen above, the turnovers of the variables and the singular vectors of **B** provide two complementary methods. The interplay between these three alternative representations can be seen in the LV of the corresponding equivalence class. The constant matrix **BA** does not result from a linearization, it defines all interactions between variables the whole phase space. Although there is no closed form for the singular/eigen-values of a matrix product, a great deal can be learned by calculating the SVD of both **A** and **B**:

(24) $$BA=U_{B}\Sigma_{B}W\Sigma_{A}V_{A}^{T}$$

where $W=V_{B}^{T}U_{A}$. The three matrices **U_B_**, **W**, and **U_A_** are unitary and will not amplify or dampen any perturbation to the variables. Any change in the norm of the perturbation will come from the two diagonal matrices of singular values, one coming from the stoichiometric component of the system, **Σ_*A*_**, and one from the kinetic **Σ_*B*_**. When only one of these matrices has extreme values it will dominate the response of the system and one of the two methods mentioned above will be accurate. No sudden changes of the jacobian are to be expected, since the Jacobian of an normalized LV system is precisely (Equation 24), see Dıaz-Sierra et al. (1999). When both sets of singular values are in the same range, the system will not be decomposable by time hierarchies. Extreme cases, where both sets of singular values have big differences, will result in systems where the time hierarchies shift along the orbits of the system. In such cases, Equation (24) would be a good starting point to identify regions of interest in the parameter and in the phase space.

### 3.5. A simple example

**Figure 1:**
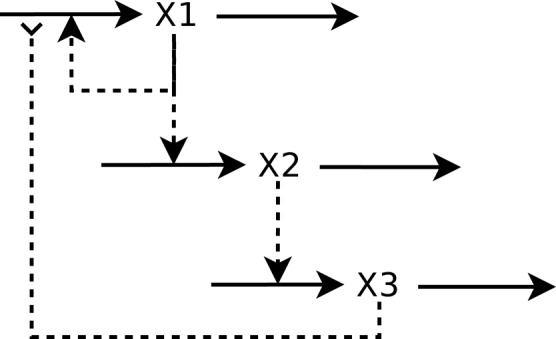
**A simple biochemical network**. Full arrowhead indicates activation and reverse arrowhead indicates inhibition.

Lets start with a model of a small regulatory network of three genes that affect one another's induction as depicted in Figure 1. Obtaining the GMA model is straightforward:

(25) $$\begin{array}{l} {\dot{x}}_{1}=\alpha_{1}x_{1}^{g_{1,1}}x_{2}^{-g_{1,3}}-\beta_{1}x_{1}\\ {\dot{x}}_{2}=\;\alpha_{2}x_{1}^{g_{2,1}}-\beta_{2}x_{2}\\ {\dot{x}}_{3}=\;\alpha_{3}x_{2}^{g_{3,2}}-\beta_{3}x_{3} \end{array}$$

which can be rewritten as a GLV by just factoring the variables out:

(26) $$\begin{array}{l} {\dot{x}}_{1}=\;x_{1}\left(\alpha_{1}x_{1}^{g_{1,1}-1}x_{2}^{-g_{1,3}}-\beta_{1}\right)\\ {\dot{x}}_{2}=x_{2}\left(\alpha_{2}x_{1}^{g_{2,1}}x_{2}^{-1}-\beta_{2}\right)\\ {\dot{x}}_{3}=\;x_{3}\left(\alpha_{3}x_{2}^{g_{3,2}}x_{3}^{-1}-\beta_{3}\right) \end{array}$$

So

(27) $$B=\left(\begin{array}{ccc} g_{11}-1 & 0 & -g_{13}\\ g_{21} & -1 & 0\\ 0 & g_{32} & -1 \end{array}\right)$$

Computing the turnovers of the variables in Equation (25) is straightforward:

$$f_{i}=\beta_{i}\;\forall i$$

normalizing:

(28) $$\begin{array}{ll} {\dot{z}}_{1}= & \;\beta_{1}(z_{1}^{g_{1,1}}z_{2}^{-g_{1,3}}-z_{1})\\ {\dot{z}}_{2}= & \;\beta_{2}(z_{1}^{g_{2,1}}-z_{2})\\ {\dot{z}}_{3}= & \;\beta_{3}(z_{2}^{g_{3,2}}-z_{3}) \end{array}$$

**Figure 2:**
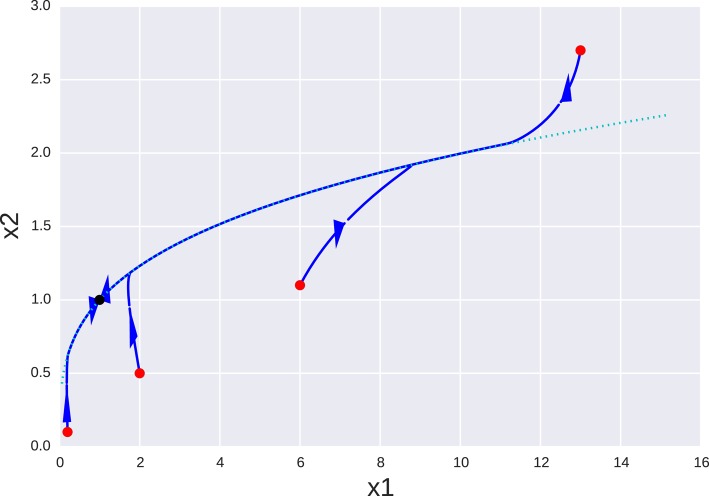
**Orbits of a simple genetic network for different initial conditions (red dots) projected onto the ***x***_**1**_ − ***x***_**2**_ plane**. Orbits clearly evolve toward a one dimensional manifold (dotted line) that follows to the singular vector *v*_3_ corresponding to the smallest singular value. The equilibrium point **x** = **1** is marked as a black dot. Red dots mark starting points of different orbits and the black dot is the equilibrium point they all tend to.

**Figure 3:**
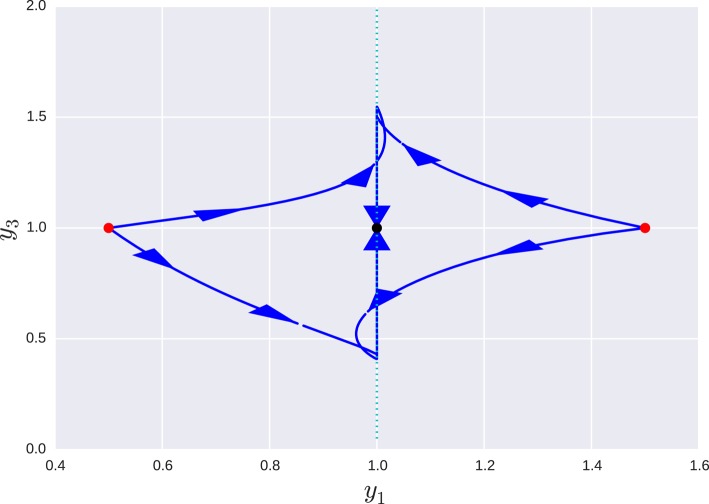
**Projection of the orbits of a the transformed system onto the ***y***_**1**_ − ***y***_**3**_ plane for different initial conditions**. In the new coordinate system *x* = *y*^*V*^, each axis correspond to a singular value, so the manifold shown in the previous figure, is now parallel to the *y*_3_ axis (dotted line). Red dots mark starting points of different orbits and the black dot is the equilibrium point they all tend to.

**Figure 4:**
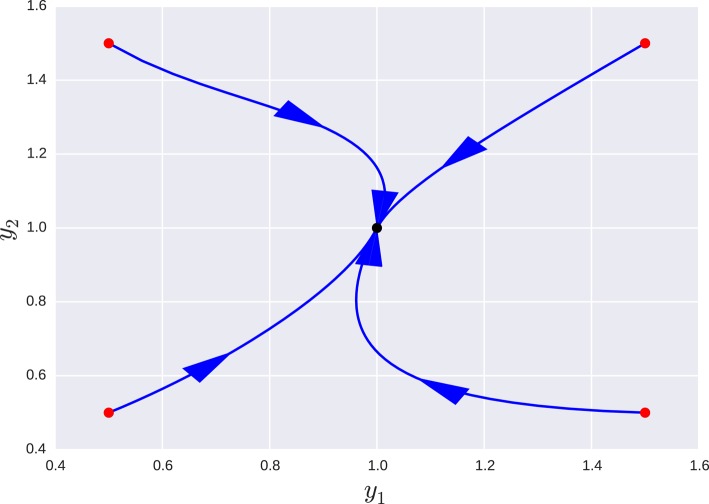
**Projection of the orbits of a the transformed system onto the ***y***_**1**_ − ***y***_**2**_ for different initial conditions**. In this projection, the manifold appears as a point aligned with the equilibrium point. Changing the values of *y*_3_ within an order of magnitude resulted in no appreciable change in this projection, since the third variable is *de facto* uncoupled. Red dots mark starting points of different orbits and the black dot is the equilibrium point they all tend to.

The Jacobian matrix is:

J=diag(β) B

for the particular case β_*i*_ = 1 ∀β all the turnover numbers are also 1 and all variables are expected to operate in the same time scale. However, as can be seen in Figure 2, where a special case is simulated—*g*_11_ = 1.1, *g*_13_ = 0.48, *g*_21_ = 0.3, *g*_32_ = 0.7—the system approaches a slow manifold defined by the singular vector with the smallest singular value of **B**: **v** = (0.939, 0.281, 0.197). So the system has a slow manifold. Transformation using Equation (22), an alternative formulation is obtained with variables (*y*_1_, *y*_2_, *y*_3_) and matrices:

(29) $$\lambda =\left(\begin{array}{c} -0.050\\ 0.99\\ -1.4 \end{array}\right)$$

(30) $$A=\left(\begin{array}{ccc} -0.10 & 0.78 & -0.62\\ 0.33 & -0.57 & -0.76\\ 0.94 & 0.28 & 0.20 \end{array}\right)$$

(31) $$B=\left(\begin{array}{ccc} 0.29 & 0.40 & -0.00056\\ -0.81 & 0.66 & 0.00019\\ 1.2 & 0.36 & 0.00027 \end{array}\right)$$

and as can be seen by the small exponents of *y*_3_, this variable has negligible influence on the dynamics of the other two, Figures 3, 4.

The same procedure can be done applying the decomposition shown in Equation (3.1) to obtain the equations for the log modes. In this case, the results are very similar to those already shown, since the Jacobian matrix of the system **J** = **B**. Even though the similarity decomposition that defined the log-modes is not equal to svd decomposition, the coefficients of the slowest log-mode of the system are within 0.5% of those of *v*_3_. Additional simulations show that special cases with well conditioned **B** led to similar time-scales using turnover numbers and log-modes, for cases with similar turnovers, the log-modes are similar to the slow manifolds predicted by **B**, as can be expected from Equation (24)—data not shown.

### 3.6. A large network

The toy model shown above is useful to understand the theory behind the methods, but in order to test the performance of the method on large scale models, randomly generated genetic networks were used. Genetic networks can be modeled using s-systems of the form ${ẋ}_{i}=\alpha_{i}\prod_{j}x_{j}^{g_{i,j}}-\beta_{i}x_{i}$, where the interactions between genes are concentrated in the kinetic orders of the positive term. The turnover can be factored out :

(32) $${\dot{x}}_{i}=F_{i}\left(\prod_{j}x_{j}^{g_{i,j}}-x_{i}\right)$$

The details of how the networks were generated are shown in the methods section, and the results were satisfactory in all cases. Here we will show the analysis of a representative network with 75 variables divided in three time scales with turnover numbers of 1, 100, and 10^4^. The number of variables in each group (time-scale) was 10, 25, and 40 respectively. The network is defined by 356 parameters: 281 non-zero kinetic orders and 75 turnover numbers. The parameters values of the s-system model are provided as supplementary data.

The existence of three time scales opens several possibilities. If the model is to be partitioned in two, the variables in the middle range can be assigned to the fast or slow subsystem. Moreover, successive separation can lead to three different submodels, one per time scale. Each approach will generate systems with different accuracy and degree of stiffness, so the optimal decision will depend on the goal of the analysis.

**Figure 5:**
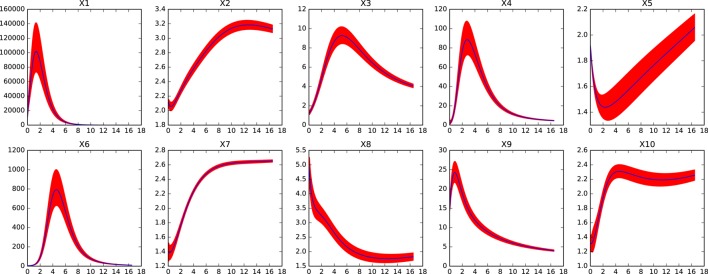
**Dynamics of ***x***_**1**_-***x***_**10**_ in the reduced system for the large genetic network model after removing variables ***x***_**11**_ to ***x***_**75**_**. Red shaded areas show the deviation of repeated simulations using the full system for different initial values of the eliminated variables. Three standard deviations above and below are shown. See text for details.

**Figure 6:**
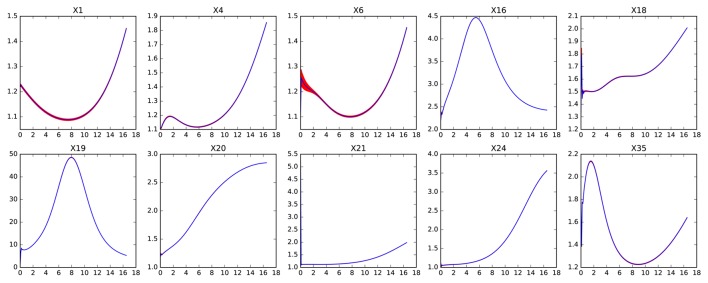
**Dynamics of the worst performing variables in the reduced system for the large genetic network model after removing variables ***x***_**36**_ to ***x***_**75**_**. Red shaded areas show the deviation of repeated simulations using the full system for different initial values of the eliminated variables. Three standard deviations above and below are shown. See text for details.

**Figure 7:**
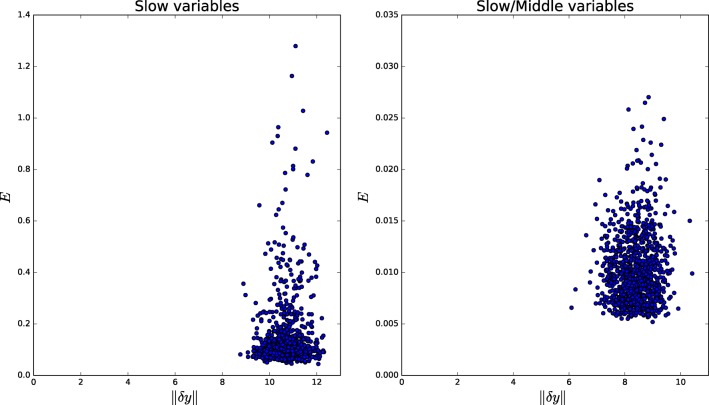
**Accumulated error for perturbations of different sizes in the genetic network model**. The **left** pane shows the error for the fully reduced model consisting only the slow variables *x*_1_ to *x*_10_. The **right** pane shows the model with the slow and middle scale variables, *x*_1_ to *x*_35_.

Figures 5, 6 show the errors in the dynamics of 100 different simulations of the two possible slow systems. In the first case, a system with only ten variables is obtained, in the second, the final number of slow variables is thirty five. Figure 7 shows the accumulated error. As can be seen, the smallest model has a much higher error but still agrees qualitatively with the dynamics of the full system. The bigger model, has an extremely small error but it still contains variables operating in two different time scales. This increases the computational cost of integration as shown in Table 1. The bigger model provides high accuracy with a substantial improvement in computational cost and a significant reduction in complexity and the number of variables. Since most biological measurements are subjected to high levels of noise, the smallest and much simpler model system will often be adequate as well.

**Table 1:** **Comparison of simulation times between original and reduced models of the large randomized networks**.

**Genetic network**		
	**Time**	**QSS**
Full	1109 ± 400	none
Slow	3.6 ± 0.2	*x*_11_ to *x*_75_
Slow/Middle	193 ± 6	*x*_36_ to *x*_75_

**Figure 8:**
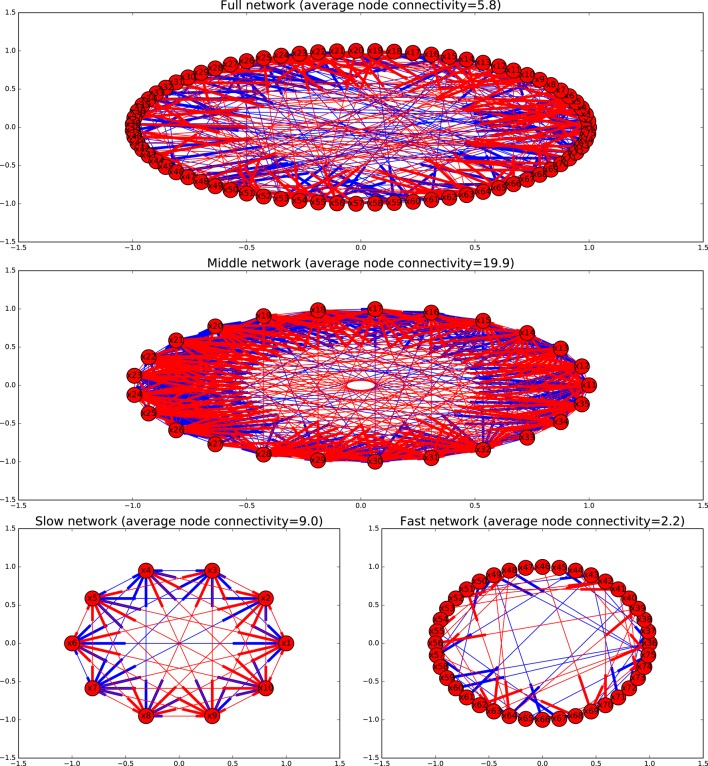
**Graphs depicting the different models as directed graphs**. Activating interactions are shown in blue, inhibitory interactions in red.

Finally, the network can be split into three different submodels able to reproduce the slow, middle and fast dynamics respectively. Figure 8 shows how the reduction processes affects the connectivity of the network. Submodels of the fast dynamics, reduce the degree of connectivity, since many connections between fast variables happen through slow variables that are frozen in the fast time scales. Submodels of slower timescales, experience the opposite effect, since the variables that are eliminated through the quasi-steady-state assumption become links between slow variables.

### 3.7. Examples from real models

In order to test the applicability to real cases, three classical s-system models from the literature (Voit, 2000) were taken as examples for benchmarking: A very simplified model for the anaerobic fermentation of *Saccharomyces cerevisiae* with 5 variables, a model for the purin metabolism in man consisting of 16 variables and one for the tricarboxylic acid cycle in *Dictyostelium discoideum* constituted by 13 variables. These three models have been also used for benchmarking an alternative method of model reduction, which will enable further comparisons.

All three models had well conditioned **B** matrices, so timescales were assigned to each variable according to their turnover number.

#### 3.7.1. Yeast

**Figure 9:**
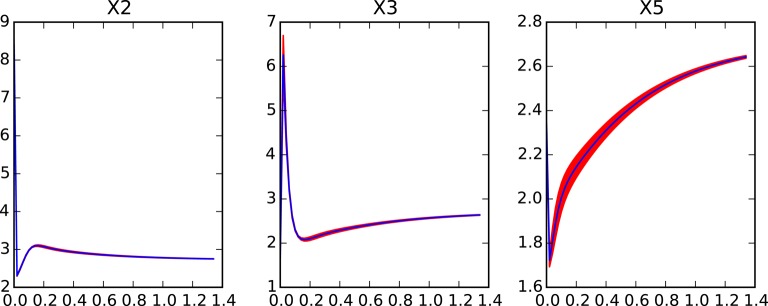
**Dynamics of the reduced system for the yeast model after removing fast variables ***x***_**1**_ and ***x***_**4**_**. Red shaded areas show the deviation of repeated simulations using the full system for different initial values of the eliminated variables. Three standard deviations above and below are shown. See text for details.

**Figure 10:**
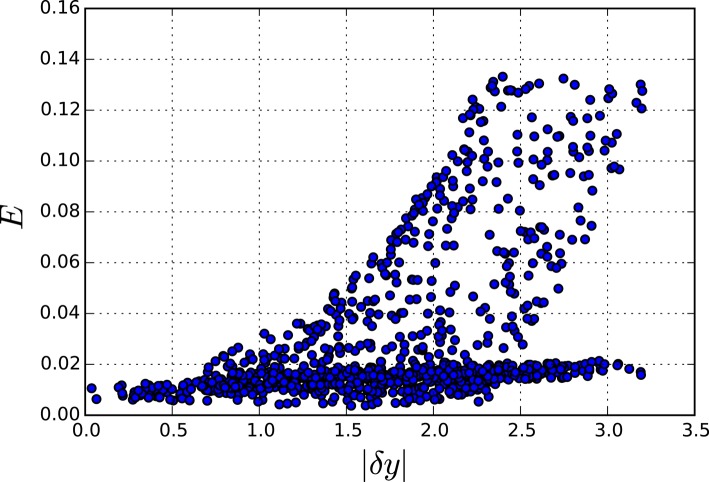
**Accumulated error for perturbations of different sizes in the reduced Yeast model**. Each point represents one simulation where the fast variables were perturbed by |dy| and the overall error E was calculated.

Eliminating the two fastest metabolites of this model of yeast glycolysis results in a robust reduced system that still can reproduce the slow dynamics with great accuracy (well within experimental error), as can be seen in Figures 9, 10. Even a perturbation δ*y* of 3 still results in less than 14% error **E**.

#### 3.7.2. TCA cycle

**Figure 11:**
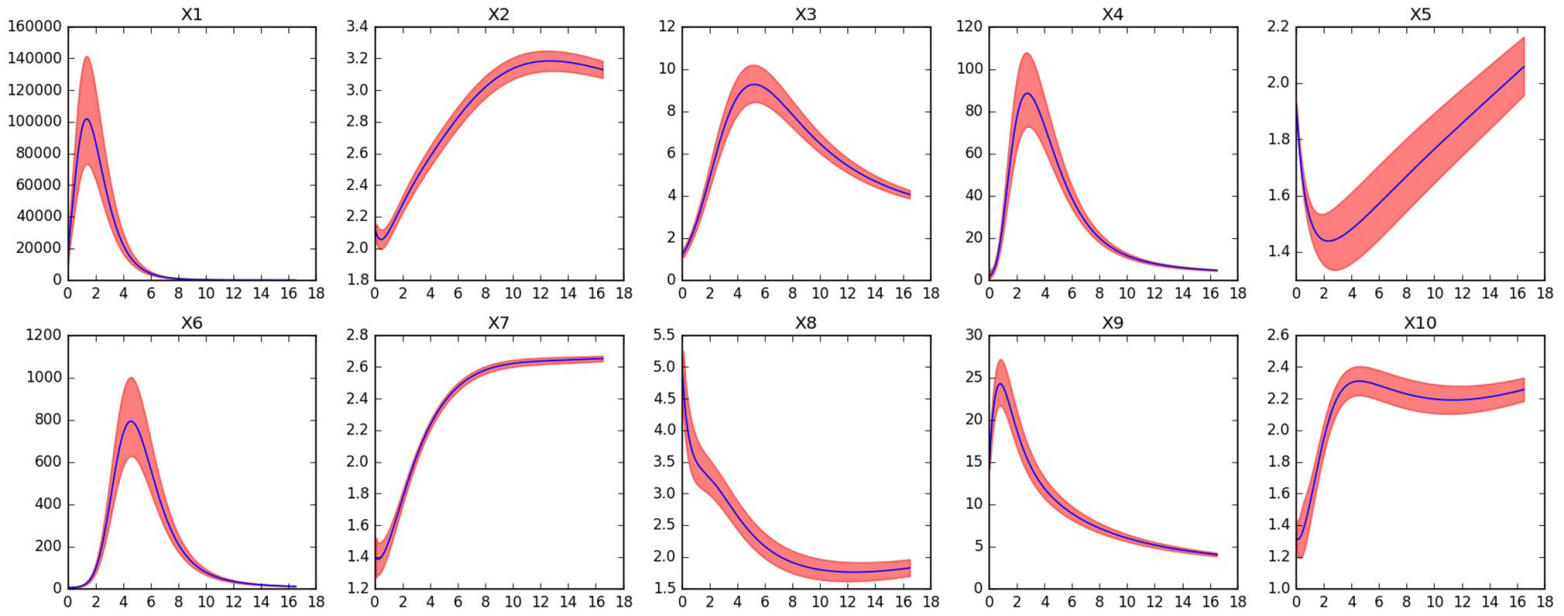
**Dynamics of the reduced system for the TCA cycle model after removing fast variables ***x***_**1**_,***x***_**2**_,***x***_**4**_,***x***_**10**_, and ***x***_**12**_**. Red shaded areas show the deviation of repeated simulations using the full system for different initial values of the eliminated variables. Three standard deviations above and below are shown. See text for details.

**Figure 12:**
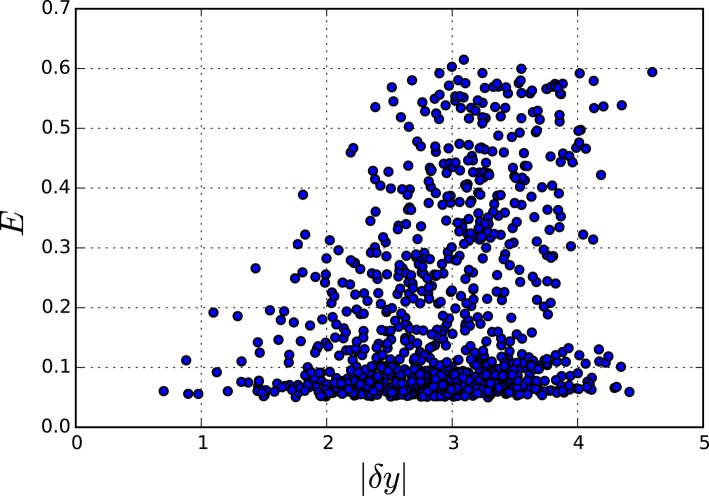
**Accumulated error for perturbations of different sizes in the reduced TCA model**. Each point represents one simulation where the fast variables were perturbed by |dy| and the overall error E was calculated.

The system is also reduced to less than two thirds of its size and results in good quantitative agreement with the full system. Figure 11 shows how some variables reproduce the dynamic perfectly while *x*_6_ and *x*_8_ go through a short adaptation phase where their dynamics are not as robust as the rest. Accumulated error is shown in Figure 12.

#### 3.7.3. Purine metabolism

**Figure 13:**
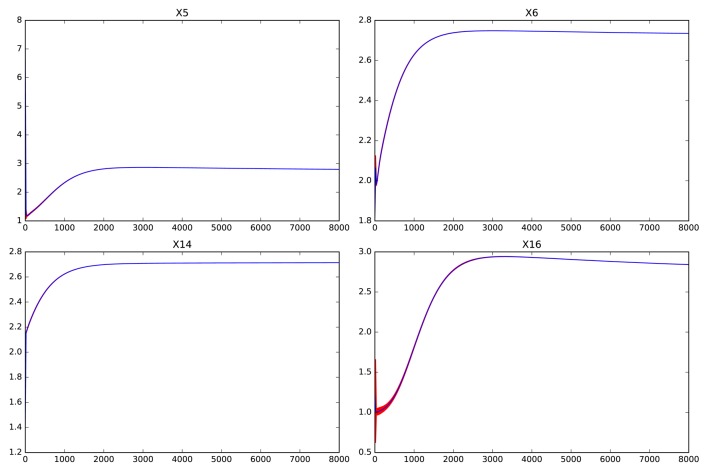
**Dynamics of ***x***_**16**_ in the reduced system for the Purines metabolism model after removing fast variables ***x***_**3**_,***x***_**8**_, and ***x***_**13**_**. Red shaded areas show the deviation of repeated simulations using the full system for different initial values of the eliminated variables. Three standard deviations above and below are shown. See text for details.

**Figure 14:**
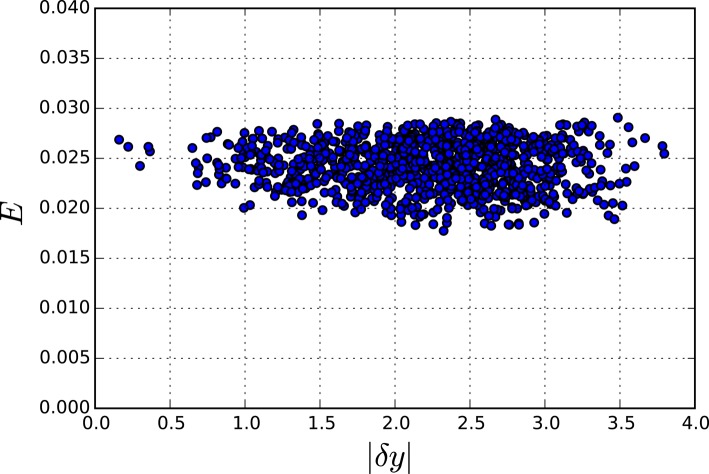
**Accumulated error for perturbations of different sizes in the reduced Purines metabolism model**. Each point represents one simulation where the fast variables were perturbed by |dy| and the overall error E was calculated.

In this example, a more conservative approach is shown, where eliminating only a small set of the total number of variables shows a great quantitative agreement between the full and the reduced systems. Figure 13 shows the only variable where an appreciable difference between the systems can be found. Accumulated error shown in Figure 14.

#### 3.7.4. Performance

Two further metrics will be considered to evaluate the performance of the method: the reduction in simulation times due to the model reduction and the amount of variables eliminated in comparison to the alternative method (Liu et al., 2013) similar method. There is, to our knowledge only one method that has exploited the regular structure of canonical models to produce a a model reduction algorithm (Liu et al., 2013). The alternative method does not provide a separation into submodels, it concerns itself exclusively with the elimination of variables using multicriteria optimization based on reactive weight, sensitivity, and flux analyses. Based on such optimization, the model is reformulated to eliminate one or more variables. For the sake of comparison, the methods presented in this study were used to obtain reduced models with total accumulated errors that were comparable to those of the previously mentioned approach, the number of variables that each method was able to remove is then compared.

Table 2 shows that model reduction always resulted in a significant improvement on the simulation times. Moreover, the number of reduced variables is always higher than or equal to the much more complex (and computationally demanding) existing method.

**Table 2:** **Comparison of simulation times between original and reduced models of the Yeast, TCA and purine models**.

	**Simulation time**		**Eliminated variables**	
	**Full model**	**Reduced model**	**This study**	**Liu et al., 2013**
Yeast	31.5 ± 1.0	11.3 ± 0.6	*x*_1_, *x*_2_, *x*_4_, *x*_5_	No reduction
TCA	71.1 ± 7.4	50.9 ± 8.2	*x*_1_, *x*_2_, *x*_4_, *x*_10_, *x*_12_	*x*_7_
Purines	492.1 ± 46.6	260.8 ± 45.7	*x*_3_, *x*_8_, *x*_13_	*x*_6_, *x*_14_, *x*_16_

## 4. Discussion

One of the bottlenecks for modeling biological systems is the need to find values for a great amount of parameters that cannot be measured directly. That, and the impossibility to predict how a change in the value of such parameters will change the dynamics of the system, limit the reliability of numerical simulations. It is therefore imperative to find reliable tools for the global analysis and model reduction for non-linear systems.

Canonical, non-linear systems are flexible enough to reproduce any kind of non-linear behavior and, at the same time, all the information defining a particular model is encoded in two matrices and a vector. Methods like recasting (Savageau and Voit, 1987; Hernández-Bermejo et al., 1998) enable to rewrite virtually any non-linear system in one of the canonical forms treated here. Moreover, Design Space Analysis (Savageau et al., 2009) enables to decompose the parameter space of any system into qualitatively similar regions, each described by an s-system.

These formalisms offer the exciting possibility of converting very abstract problems into simple linear algebra operations. Converting topologically equivalent systems into one another is done with three simple matrix products and identifying a slow manifold can be done by Singular Value Decomposition. Moreover, any model in one of these formalisms can be exactly converted into a set of Lotka–Volterra equations. In the Lotka–Volterra representation, a single constant matrix determines the interactions between variables for the whole phase space, as opposed to a linearization, where the constant matrix is merely a local representation in the vicinity of an equilibrium. Decomposing this matrix into its kinetic and stoichiometric parts, provides a great deal of insight into the structure of the system through the examination of the two corresponding sets of singular values. These results obtained with simple linear algebra, are as good as those that can be obtained using much more complicated approaches (Liu et al., 2013) as well as more general. The significance of this can best be appreciated in light of an example attributed to professor Grötschel (Holdren et al., 2010), a certain linear programming problem that would take 82 years to be solved by a computer in 1988 would be solved in roughly a minute by a modern computer 15 years later. Of this improvement by a factor of 43 million, 1000 could be attributed to hardware improvements and the remaining 43,000 to improvements in numerical algorithms, mostly numerical linear algebra.

## Author contributions

AK and AM proposed the topic and guided the research. HL and AM developed the concept and carried out the simulations and data analysis. All authors participated in the writing of the manuscript and approved the final version.

### Conflict of interest statement

The authors declare that the research was conducted in the absence of any commercial or financial relationships that could be construed as a potential conflict of interest.
