# Supplementary material for: Time Hierarchies and Model Reduction in Canonical Non-linear Models
Source: Front Genet. 2016 Sep 21;7:166. doi: 10.3389/fgene.2016.00166 (PMC5030239; doi:10.3389/fgene.2016.00166)
Supplement: Supplementary file 1 [file DataSheet1.PDF]

frontiers

Supplementary Material: Time hierarchies and model reduction in canonical non-linear models

Hannes Löwe,[1], Andreas Kremling[1] and Alberto Marin-Sanguino[1,*]
*Correspondence:
Corresponding Author
A.Marin@lrz.tu-muenchen.de

1 SUPPLEMENTARY DATA

1 APPENDIX:DETAILED CALCULATIONS

1.1 Direct notation

The structural regularity of power law systems enables to use a particular notation that greatly simplifies algebraic manipulations. For that reason, a direct notation was introduced in Voit (1991) which has been recently adapted and is starting to become common Marin-Sanguino et al. (2010); Müller and Regensburger (2014). This notation starts by defining the power law as a matrix function. The key property that enables such a definition is that power laws become linear by taking logarithms:

$$\ln q = \ln \left(x_1^{a_1} \, x_2^{a_2} \, \cdots \, x_k^{a_k} \right) = a_1 \, \ln x_1 \, + \, a_n \, \ln x_2 \, + \, \cdots \, + \, a_k \, \ln x_k$$

so with help of the element-wise logarithm of a matrix (or vector) we can generalize expressions like the one above as:

$$\ln \mathbf{q} = \mathbf{A} \ln \mathbf{x}$$

where an $m \times n$ matrix $\mathbf{A}$ and a $n \times 1$ vector $\mathbf{x}$ would lead to a $m \times 1$ vector $\mathbf{q}$.

Now, ust as we can define an exponential of base a in the scalar case:

$$x^a = \left(e^{\ln x} \right)^a = e^{a \ln x} \;\; \forall \, x > 0 \tag{1}$$

We can define exponentials with matrices as base and exponent:

$$\mathbf{x}^{\mathbf{A}} = \exp \left(\ln \mathbf{x}^{\mathbf{A}} \right) = \exp \left(\mathbf{A} \ln \mathbf{x} \right) \;\; \forall \, \mathbf{x} > 0 \tag{2}$$

where $\exp()$ is used elementwise, just as the logarithm before. This function is called S-exponentialVoit (1991).

$$\mathbf{x}^{\mathbf{0}} = \mathbf{1} \tag{3}$$

where $\mathbf{0}$ is a matrix of zeros and $\mathbf{1}$ a vector (or a matrix) of ones.

$$\mathbf{x}^{\mathbf{I}} = \mathbf{x} \tag{4}$$

Scalar exponentials and logarithms find their way to many applications because they link products and sums in a very convenient way. The the s-exponential offers similar properties. For instance, the composition:

$$\left(\mathbf{x}^{\mathbf{A}}\right)^{\mathbf{B}} = \mathbf{x}^{\mathbf{B}\cdot\mathbf{A}} \tag{5}$$

But unlike scalars, for which a unique product is defined, matrices can be multiplied in different ways. This diversity manifests itself in the properties of the s-exponential, some of which are linked to alternative matrix products.

$$\mathbf{x}^{\mathbf{A}+\mathbf{B}} = \mathbf{x}^{\mathbf{A}} \circ \mathbf{x}^{\mathbf{B}} \tag{6}$$

here $\circ$ is the Hadamard or element-wise product. this product is commutative and always takes precedence over the matrix product The s-exponential, offers a very convenient formulation for the inverse element of the Hadamard product:

$$\mathbf{A} \circ \mathbf{A}^{-\mathbf{I}} = \mathbf{1} \tag{7}$$

since $\mathbf{A}^{-\mathbf{I}}$ is a matrix of recipricals $\frac{1}{a_{i,j}}$ and $\mathbf{1}$ is the identity element of the Hadamard product. This provides a convenient shorthand when we want to divide some power-laws by their variables, let's say $q_i = x_1^{a_1}\, x_2^{a_2}\, \cdots\, x_k^{a_k}/x_i$ in direct notation:

$$\mathbf{q} \circ \mathbf{x}^{-\mathbf{I}} = \mathbf{x}^{\mathbf{A}} \circ \mathbf{x}^{-\mathbf{I}} = \mathbf{x}^{\mathbf{A}-\mathbf{I}} \tag{8}$$

As a way to help getting used to the notation lets transform the equations of an s-system to logarithmic coordinates $\mathbf{y} = \ln \mathbf{x}$:

$$\dot{\mathbf{y}} = \dot{\mathbf{x}} \circ \mathbf{x}^{-\mathbf{I}} = \left(\alpha \circ \mathbf{x}^{\mathbf{G}} - \beta \circ \mathbf{x}^{\mathbf{H}}\right) \circ \mathbf{x}^{-\mathbf{I}} = \alpha \circ \mathbf{x}^{\mathbf{G}-\mathbf{I}} - \beta \circ \mathbf{x}^{\mathbf{H}-\mathbf{I}} \tag{9}$$

and from the definition of the s-exponential, we can remove $\mathbf{x}$ completely:

$$\dot{\mathbf{y}} = \alpha \circ \exp\left((\mathbf{G}-\mathbf{I})\ \mathbf{y}\right) - \beta \circ \exp\left((\mathbf{H}-\mathbf{I})\ \mathbf{y}\right) \tag{10}$$

which is the known logarithmic representation of s-systems.There is a convenient property of the hadamard product of two vectors that permits switching between the two different kinds of matrix product. Let's define matrix, $\text{diag}(\mathbf{x})$, as a matrix whose entries are zero except for the main diagonal, which contains the elements of vector $\mathbf{x}$. Then,

$$\mathbf{x} \circ \mathbf{y} = \text{diag}(\mathbf{x})\, \mathbf{y} = \mathbf{x}\, \text{diag}(\mathbf{y}) \tag{11}$$

This would for instance allow to remove the Hadamard products from the above equation:

$$\dot{\mathbf{y}} = \text{diag}(\alpha)\ \exp\left((\mathbf{G}-\mathbf{I})\ \mathbf{y}\right) - \text{diag}(\beta)\ \exp\left((\mathbf{H}-\mathbf{I})\ \mathbf{y}\right) \tag{12}$$

and choose the kind of product that is most convenient on each ocassion.

Because of the isomorphism between the matrix exponential and matrix product shown in equation 2. Operations based on block matrices also apply to expressions with s-exponentials. For instance, the

variables vector $\mathbf{x}$ is often partitioned into dependent and independent variables $\mathbf{x} = (\mathbf{x_d}\ \mathbf{x_i})^T$, combining the properties seen above we can write:

$$\mathbf{x}^{\mathbf{G}} = \begin{pmatrix} \mathbf{x_d} \\ \mathbf{x_i} \end{pmatrix}^{(\mathbf{G_d}\ \mathbf{G_i})} = \mathbf{x_d}^{\mathbf{G_d}} \circ \mathbf{x_i}^{\mathbf{G_i}} \tag{13}$$

this relation will be extremely useful to operate with partitions between fast and slow variables below.

Besides being convenient to rewrite power-law equations in different forms, the s-exponential can be differentiated,

$$\mathbf{D}\left(\mathbf{x}^{\mathbf{G}}\right) = \mathrm{diag}(\mathbf{x}^{\mathbf{G}})\,\mathbf{G}\,\mathrm{diag}^{-1}(\mathbf{x}) \tag{14}$$

1.2 Non-dimensionalization

$$\dot{\mathbf{x}}_{\mathbf{d}} = \alpha \circ \mathbf{x_d}^{\mathbf{G_d}} \circ \mathbf{x_i}^{\mathbf{G_i}} - \beta \circ \mathbf{x_d}^{\mathbf{H_d}} \circ \mathbf{x_i}^{\mathbf{H_i}} \tag{15}$$

Making the change of variables:

$$\mathbf{x_d} = |\mathbf{x_d}|_0 \circ \mathbf{z}$$

where $|\mathbf{x_d}|_0$ is the steady state value of $\mathbf{x_d}$.

$$\dot{\mathbf{z}} = |\mathbf{x_d}|_0^{-\mathbf{I}} \left(\alpha \circ |\mathbf{x_d}|_0^{\mathbf{G_d}} \circ \mathbf{z}^{\mathbf{G_d}} \circ \mathbf{x_i}^{\mathbf{G_i}} - \beta \circ |\mathbf{x_d}|_0^{\mathbf{H_d}} \circ \mathbf{z}^{\mathbf{H_d}} \circ \mathbf{x_i}^{\mathbf{H_i}}\right)$$

By definition:

$$\alpha \circ |\mathbf{x_d}|_0^{\mathbf{G_d}} \circ \mathbf{x_i}^{\mathbf{G_i}} = \beta \circ |\mathbf{x_d}|_0^{\mathbf{H_d}} \circ \mathbf{x_i}^{\mathbf{H_i}}$$

so

$$\dot{\mathbf{z}} = \mathbf{f} \circ \left(\mathbf{z}^{\mathbf{G_d}} - \mathbf{z}^{\mathbf{H_d}}\right) \tag{16}$$

where:

$$\mathbf{f} = \alpha \circ |\mathbf{x_d}|_0^{\mathbf{G_d}-\mathbf{I}} \circ \mathbf{x_i}^{\mathbf{G_i}} = \beta \circ |\mathbf{x_d}|_0^{\mathbf{H_d}-\mathbf{I}} \circ \mathbf{x_i}^{\mathbf{H_i}}$$

since:

$$|\mathbf{x_d}|_0 = \left(\alpha^{-\mathbf{I}} \circ \beta\right)^{\mathbf{A}_d^{-1}} \circ \mathbf{x_i}^{-\mathbf{A}_d^{-1}\mathbf{A}_i}$$

$$\mathbf{f} = \alpha^{\mathbf{I}-(\mathbf{G}_d-\mathbf{I})\mathbf{A}_d^{-1}} \circ \beta^{(\mathbf{G}_d-\mathbf{I})\mathbf{A}_d^{-1}} \circ \mathbf{x_i}^{\mathbf{G}_i-(\mathbf{G}_d-\mathbf{I})\mathbf{A}_d^{-1}\mathbf{A}_i} \tag{17}$$

each component of f will be multiplying an equation and therefore define its time-scale.

Now if the equations in 16 are ordered according to their f-factor in decreasing order, the variables can be classified as slow or fast by finding a variable x_k such that $\|f_{k+1} - f_k\|$ is maximal. Now a non-dimensionalization for time can be applied $\tau = f_k\, t$

$$\frac{d\mathbf{z}}{d\hat{t}} = \frac{1}{f_k}\mathbf{f} \circ \left(\mathbf{z}^{\mathbf{G_d}} - \mathbf{z}^{\mathbf{H_d}}\right)$$

defining $\varepsilon = \frac{f_{k+1}}{f_k}$ the factor multiplying the equations will be: $f_1/f_{k+1}, \dots \varepsilon, 1, \dots, f_n/f_{k+1}$. Now the variables can be partitioned in two vectors $\mathbf{z_S} = (z_1, \dots, z_k)$ and $\mathbf{z_F} = (z_{k+1}, \dots, z_n)$. After partitioning the parameter vectors and matrices appropriately and factoring ε out of the equations:

$$\begin{aligned}\frac{d\mathbf{z_S}}{d\hat{t}} &= \varepsilon\, \hat{\mathbf{f}}_\mathbf{S} \circ \left(\mathbf{z_S}^{\mathbf{G_{S,S}}} \circ \mathbf{z_F}^{\mathbf{G_{S,F}}} - \mathbf{z_S}^{\mathbf{H_{S,S}}} \circ \mathbf{z_F}^{\mathbf{H_{S,F}}}\right) \\ \frac{d\mathbf{z_F}}{d\hat{t}} &= \hat{\mathbf{f}}_\mathbf{F} \circ \left(\mathbf{z_S}^{\mathbf{G_{F,S}}} \circ \mathbf{z_F}^{\mathbf{G_{F,F}}} - \mathbf{z_S}^{\mathbf{H_{F,S}}} \circ \mathbf{z_F}^{\mathbf{H_{F,F}}}\right)\end{aligned} \tag{18}$$

where $\hat{f}_{S,i} = f_i/f_k < 1$ and $\hat{f}_{F,i} = f_i/f_{k+1} > 1$.

1.3 Fast dynamics: inner solution

$$\begin{aligned}\dot{\mathbf{x}}_\mathbf{S} &= \alpha_\mathbf{S} \circ \mathbf{x_S}^{\mathbf{G_{S,S}}} \circ \mathbf{x_F}^{\mathbf{G_{S,F}}} \circ \mathbf{x_i}^{\mathbf{G_{S,i}}} - \beta_\mathbf{S} \circ \mathbf{x_S}^{\mathbf{H_{S,S}}} \circ \mathbf{x_F}^{\mathbf{H_{S,F}}} \circ \mathbf{x_i}^{\mathbf{H_{S,i}}} \\ \dot{\mathbf{x}}_\mathbf{F} &= \alpha_\mathbf{F} \circ \mathbf{x_S}^{\mathbf{G_{F,S}}} \circ \mathbf{x_F}^{\mathbf{G_{F,F}}} \circ \mathbf{x_i}^{\mathbf{G_{F,i}}} - \beta_\mathbf{F} \circ \mathbf{x_S}^{\mathbf{H_{F,S}}} \circ \mathbf{x_F}^{\mathbf{H_{F,F}}} \circ \mathbf{x_i}^{\mathbf{H_{F,i}}}\end{aligned} \tag{19}$$

So when $\dot{\mathbf{x}}_\mathbf{S} = 0$, the system reduces to:

$$\dot{\mathbf{x}}_\mathbf{F} = \bar{\alpha}_\mathbf{F} \circ \mathbf{x_F}^{\mathbf{G_{F,F}}} \circ \mathbf{x_i}^{\mathbf{G_{F,i}}} - \bar{\beta}_\mathbf{F} \circ \mathbf{x_F}^{\mathbf{H_{F,F}}} \circ \mathbf{x_i}^{\mathbf{H_{F,i}}} \tag{20}$$

$$\begin{aligned}\bar{\alpha}_\mathbf{F} &= \alpha_\mathbf{F} \circ \mathbf{x_S}(0)^{\mathbf{G_{F,S}}} \\ \bar{\beta}_\mathbf{F} &= \beta_\mathbf{F} \circ \mathbf{x_S}(0)^{\mathbf{H_{F,S}}}\end{aligned} \tag{21}$$

1.4 Slow dynamics: outer solution

$$\begin{aligned}\dot{\mathbf{x}}_\mathbf{S} &= \alpha_\mathbf{S} \circ \mathbf{x_S}^{\mathbf{G_{S,S}}} \circ \mathbf{x_F}^{\mathbf{G_{S,F}}} \circ \mathbf{x_i}^{\mathbf{G_{S,i}}} - \beta_\mathbf{S} \circ \mathbf{x_S}^{\mathbf{H_{S,S}}} \circ \mathbf{x_F}^{\mathbf{H_{S,F}}} \circ \mathbf{x_i}^{\mathbf{H_{S,i}}} \\ 0 &= \alpha_\mathbf{F} \circ \mathbf{x_S}^{\mathbf{G_{F,S}}} \circ \mathbf{x_F}^{\mathbf{G_{F,F}}} \circ \mathbf{x_i}^{\mathbf{G_{F,i}}} - \beta_\mathbf{F} \circ \mathbf{x_S}^{\mathbf{H_{F,S}}} \circ \mathbf{x_F}^{\mathbf{H_{F,F}}} \circ \mathbf{x_i}^{\mathbf{H_{F,i}}}\end{aligned} \tag{22}$$

So when $\varepsilon = 0$ and $|\mathbf{A_{F,F}}| \neq 0$,

$$|\mathbf{x_F}|_{qss} = \left(\alpha_\mathbf{F}^{-\mathbf{I}} \circ \beta_\mathbf{F}\right)^{\mathbf{A_{F,F}}^{-1}} \circ \mathbf{x_S}^{-\mathbf{A_{F,F}}^{-1}\mathbf{A_{F,S}}} \circ \mathbf{x_i}^{-\mathbf{A_{F,F}}^{-1}\mathbf{A_{F,i}}}$$

which can be substituted in 22 resulting in the reduced s-system:

$$\dot{\mathbf{x}}_\mathbf{S} = \hat{\alpha} \circ \mathbf{x_S}^{\hat{\mathbf{G}}_\mathbf{S}} \circ \mathbf{x_i}^{\hat{\mathbf{G}}_\mathbf{i}} - \hat{\beta} \circ \mathbf{x_S}^{\hat{\mathbf{H}}_\mathbf{S}} \circ \mathbf{x_i}^{\hat{\mathbf{H}}_\mathbf{i}} \tag{23}$$

where

$$\begin{aligned}
\hat{\alpha}_{\mathbf{S}} &= \alpha_{\mathbf{S}} \circ \left(\alpha_{\mathbf{F}}{}^{-\mathbf{I}} \circ \beta_{\mathbf{F}}\right)^{\mathbf{G}_{\mathbf{S},\mathbf{F}}\,\mathbf{A}_{F,F}^{-1}} \\
\hat{\beta}_{\mathbf{S}} &= \beta_{\mathbf{S}} \circ \left(\alpha_{\mathbf{F}}{}^{-\mathbf{I}} \circ \beta_{\mathbf{F}}\right)^{\mathbf{H}_{\mathbf{S},\mathbf{F}}\,\mathbf{A}_{F,F}^{-1}} \\
\hat{\mathbf{G}}_{\mathbf{S}} &= \mathbf{G}_{\mathbf{S},\mathbf{S}} - \mathbf{G}_{\mathbf{S},\mathbf{F}}\,\mathbf{A}_{\mathbf{F},\mathbf{F}}^{-\mathbf{1}}\,\mathbf{A}_{\mathbf{F},\mathbf{S}} \\
\hat{\mathbf{G}}_{\mathbf{i}} &= \mathbf{G}_{\mathbf{S},\mathbf{i}} - \mathbf{G}_{\mathbf{S},\mathbf{F}}\,\mathbf{A}_{\mathbf{F},\mathbf{F}}^{-\mathbf{1}}\,\mathbf{A}_{\mathbf{F},\mathbf{i}} \\
\hat{\mathbf{H}}_{\mathbf{S}} &= \mathbf{H}_{\mathbf{S},\mathbf{S}} - \mathbf{H}_{\mathbf{S},\mathbf{F}}\,\mathbf{A}_{\mathbf{F},\mathbf{F}}^{-\mathbf{1}}\,\mathbf{A}_{\mathbf{F},\mathbf{S}} \\
\hat{\mathbf{H}}_{\mathbf{i}} &= \mathbf{H}_{\mathbf{S},\mathbf{i}} - \mathbf{H}_{\mathbf{S},\mathbf{F}}\,\mathbf{A}_{\mathbf{F},\mathbf{F}}^{-\mathbf{1}}\,\mathbf{A}_{\mathbf{F},\mathbf{i}} \qquad (24)
\end{aligned}$$

REFERENCES

Voit E, editor. *Canonical Nonlinear Modeling: S-System Approach to Understanding Complexity* (Van Nostrand Reinhold. New York. US), chap. A qualitative analysis of s-systems: Hopf bifurcations (1991).

Marin-Sanguino A, Mendoza ER, Voit EO. Flux duality in nonlinear gma systems: Implications for metabolic engineering. *Journal of biotechnology* **149** (2010) 166–172.

Müller S, Regensburger G. Generalized mass-action systems and positive solutions of polynomial equations with real and symbolic exponents. *arXiv preprint arXiv:1406.6587* (2014).
